# Preventing chylous ascites after right hemicolectomy with D3 extended mesenterectomy

**DOI:** 10.1007/s00423-020-01938-4

**Published:** 2020-07-20

**Authors:** Elin Edda Seland Agustsdottir, Bojan V. Stimec, Tine Tveit Stroemmen, Ariba Ehsan Sheikh, Ilackiya Elaiyarajah, Jonas Christoffer Lindstroem, Dejan Ignjatovic

**Affiliations:** 1 Department of Digestive Surgery, Akershus University Hospital, Lørenskog, 1478 Norway; 2 Anatomy Sector, Teaching Unit, Faculty of Medicine, University of Geneva, Geneva, Switzerland; 3 Health Services Research Unit, Akershus University Hospital, Lorenskog, Norway; 4 Institute of Clinical Medicine, University of Oslo, Oslo, Norway

**Keywords:** Chylous ascites, D3 extended mesenterectomy, Fat-reduced diet, Fat-free diet, Lymphatic leak

## Abstract

**Background:**

We aim to find the incidence of chylous ascites in patients undergoing D3 extended mesenterectomy and evaluate if a routine fat-reduced diet (FRD) has a prophylactic effect.

**Methods:**

Data from 138 patients included in a D3 extended mesenterectomy trial were collected prospectively. Surgical drains and biochemical testing of drain fluid were used to find the incidence of chylous ascites among the first 39 patients, and a prophylactic fat-reduced diet was then implemented in the next 99 patients as a prophylactic measure.

**Results:**

In the first 39 patients, we found that 16 (41.0%) developed chylous ascites. After the fat-reduced diet was implemented, 1 (1.0%) of 99 patients developed chylous ascites. Drain discharge was 150 vs. 80 mL daily, respectively, and a regression analysis shows the average leakage in the group with fat-reduced diet was 105 mL/day less than in the patients with no dietary restrictions (*p* < 0.001). There were no significant differences in the rate of other complications (Fisher exact test, one-tailed *p* = 0.8845), and although there was a tendency to a shorter hospital stay when given a fat-reduced diet (7.3 ± 5.4 vs. 8.9 ± 4.9 days), the difference was not significant (*p* = 0.19).

**Conclusions:**

Chylous ascites is a very common postoperative occurrence after right colectomy with extended D3 mesenterectomy and may be prevented using a routine fat-reduced diet.

## Introduction

Small bowel and right colonic neoplasms tend to spread to the central mesentery via the lymph flow. Higher awareness of this spreading pattern inevitably leads to more extensive surgery. Performing D3 lymph node dissection in the root of the mesentery can lead to injury of the intestinal trunks and subsequent chylous ascites [1]—a well-known but traditionally uncommon complication after surgery.

D3 extended mesenterectomy entails the removal of all fatty tissue from the predefined D3 area [2], made possible and safe through preoperative 3D multi-detector computed tomography (MDCT) vascular anatomy reconstruction [3, 4]. Literature suggests that the incidence of chylous ascites after surgery, i.e., urological, gynecological, vascular, and liver (non-intestinal lymph), is estimated to be between 0.17 and 7.0% [5], while the incidence rates may be higher after gastric, colorectal, pancreatic, and small bowel surgery (intestinal lymph) with a rate between 1.0 and 11.8% [1, 5, 6]. Reports on the incidence rates of chylous ascites after D3 extended mesenterectomy are scarce in the literature. Chylous ascites occurring after bowel surgery is of a different origin when compared with chylous ascites occurring after other types of abdominal surgery. The lymph flow from the intestines is a function of dietary fat, implying that a lower intake of fat results in less lymph flow. Fat-free diet or total parenteral nutrition (TPN) is traditionally used as conservative treatment for chylous ascites [7, 8].

The aim of this article is to determine the incidence of chylous ascites in patients undergoing right colectomy with extended D3 mesenterectomy, and then evaluate the effect of a routine fat-reduced diet (FRD) 3 days after surgery.

## Material and methods

The subjects analyzed in this article are patients included into the “Safe D3 right hemicolectomy for cancer through multi-detector computed tomography (MDCT) angiography” trial (Regional Ethical Committee approval REK Sør-Øst No. 2010/3354) and registered at clinicaltrials.gov (NCT01351714). Patients were required to sign an informed consent form prior to inclusion. The trial includes mandatory preoperative 3D reconstruction of the vascular anatomy, a standardized definition of the D3 volume, a standardized surgical approach to the central (level of dissection III) lymph nodes, and a separate histopathological analysis of the D3 volume. Data collection is prospective. All these data points have been previously published [2–4, 9–11] but will be addressed in short below. While the regional ethical committee approval allows any mode of access (open [10], laparoscopic [9], and robotic assisted), the study started out through open access while laparoscopic and robotic-assisted surgery were introduced at a later point.

The question of chylous ascites was raised after a number of patients in the mentioned trial had undergone surgery, some of which developed CA postoperatively. The awareness of the relevance of chylous ascites after D3 surgery prompted a change in the trial protocol, and the change regarding the introduction of a routine fat-reduced diet as part of the study was submitted to the Regional Ethical Committee.

### The patients

Consecutive patients operated with right colectomy and D3 extended mesenterectomy at Akershus University Hospital for right-sided colon cancer from September 2014 to January 2017 were prospectively evaluated. Medical records were collected from the electronic medical record systems DIPS (Copyright 1995–2016 DIPS ASA version 7.395) and Panorama (anesthesiology electronic record system included in DIPS). Two surgeons operated all patients.

### Preoperative 3D reconstruction of the vascular anatomy

**Fig. 1:**
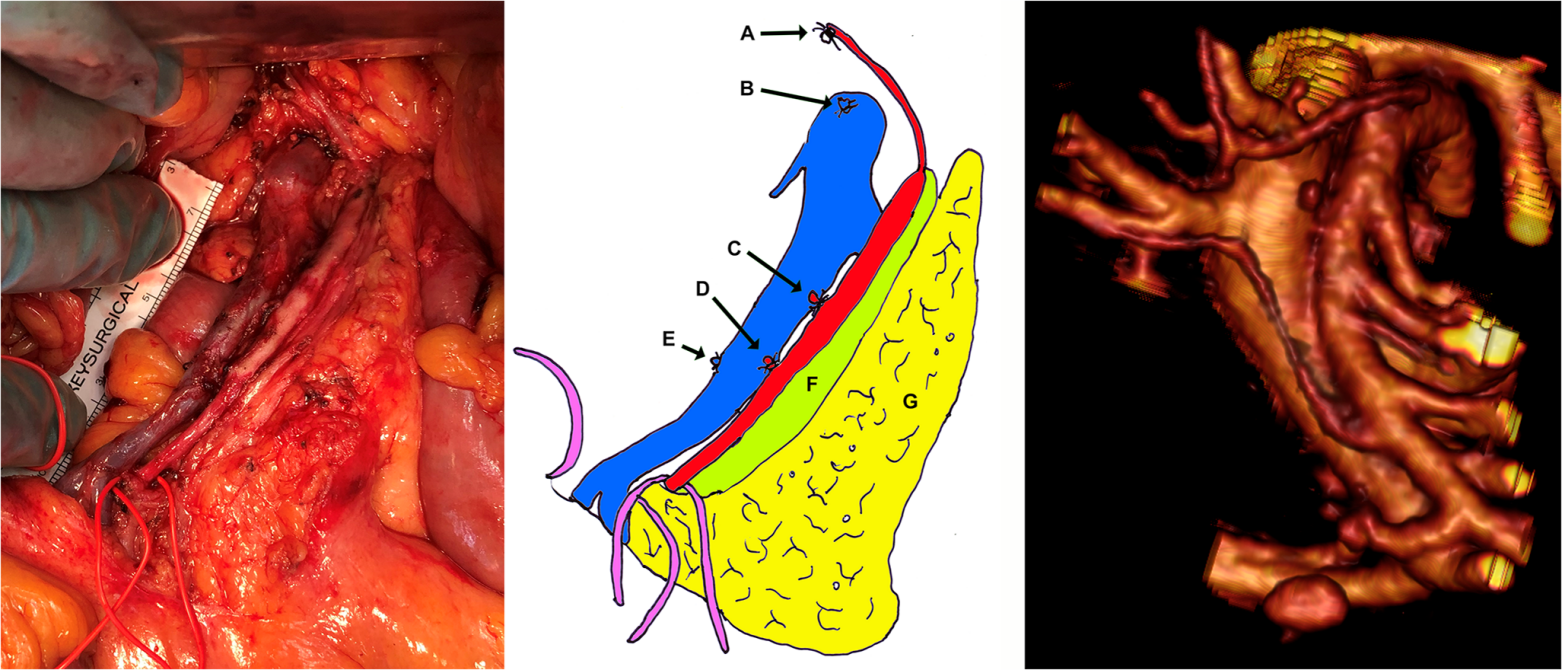
Left to right, operative image of the thoroughly dissected D3 area, schema of the operative image, and preoperative 3DVR of the same patient. a, tied right branch of the middle colic artery; b, tied middle colic vein; c, tied right colic artery; d, tied ileocolic artery; e, tied ileocolic vein; f, resected vascular sheath of the superior mesenteric artery; g, cut thickness of the mesenteric anterior leaf overlying the D3 area

Every patient included in the study had a personalized reconstruction of the vascular anatomy in the central mesentery derived from the preoperative staging CT dataset (Fig. 1). Segmentation is manually performed by BVS using the FDA-approved Osirix MD v. 10.0.2 64-bit image processing application (Pixmeo, Bernex, Switzerland), Mimics medical image processing software ver. 21.0.0.406, and 3-matic medical software ver. 13.0.0.188, both Windows 7 ultimate edition × 64 2018 (both from Materialize NV, Leuven, Belgium).

### Definition of the D3 volume

The third level of dissection (D3) is defined as the volume of tissue limited by four lines connecting anatomical landmarks (vessel origins/confluences). The cranial border lies 5 mm proximal and parallel to the line connecting the confluence of the gastrocolic trunk of Henle and the origin of the middle colic artery (MCA). The medial border lies on the left side of the superior mesenteric artery (SMA). The caudal border is 10 mm distal to the line connecting the ileocolic artery origin (ICA) and ileocolic vein confluence. The lateral border is placed 10 mm parallel to the right-hand side of the superior mesenteric vein (SMV) [2, 3]. This definition was consistently used for postoperative division of the surgical specimen into the respective D3/D2 volumes.

### Surgical approach to the central (level of dissection III) lymph nodes

The surgical technique has been previously described and encompasses removal of all fatty tissue surrounding the superior mesenteric vessels *en bloc* with the surgical specimen [4, 10]. Figure 1 depicts the preoperative 3D image (right) and the dissected D3 area at surgery in the same patient. Notice the vascular sheath of the superior mesenteric artery.

### Patient groups

Consecutive patients included in the “Safe D3 right hemicolectomy for cancer through multi-detector computed tomography (MDCT) angiography” trial were divided into two distinct chronological groups (diagnostic and treatment groups). Group 1 (diagnostic group) consists of patients where surgical drains were placed at the time of surgery and biochemical testing was performed on the drain fluid (the volume registered and fluid tested for triglyceride and cholesterol levels) in order to diagnose CA. There were no dietary restrictions, and the diet was not documented.

Group 2 (treatment group) consists of patients after the incidence of chylous ascites was established and the intervention introduced; a routine FRD to all patients operated and lasting for at least three postoperative days. If chylous ascites developed the FRD was continued until the CA was resolved. Surgical drains were routinely placed and the volume of drain discharge was registered and drain fluid was tested for triglyceride and cholesterol levels.

### Chylous ascites

The presence of milky, non-infectious discharge through the abdominal drain with triglyceride levels higher than 1.3 mmol/L and lower cholesterol levels than blood, was defined as chylous ascites. Blood triglyceride and cholesterol levels were taken only on the first postoperative day. The level of triglyceride and cholesterol in the drain discharge was analyzed on the morning of the first-, second- and third postoperative days and prolonged when needed. If the analyses did not raise suspicion of chylous ascites, the abdominal drain was removed on day 3. Volume of drain discharge was registered at 24-h intervals by nurses and noted in the electronic patient journal.

### The fat reduced diet

In cooperation with a clinical nutritionist, a FRD was compiled and thereafter introduced as a prophylactic measure. An information pamphlet was made for staff and patients to secure equal treatment. This FRD is not fat free, but the amount of fat is reduced to 10–18 g daily depending on the patients’ nutritional requirements. Recommended duration of the intervention was 3 days, and after drain removal, fat was reintroduced into the diet. In the case of chylous ascites, the FRD was not discontinued until resolution.

### Statistics

Regression modeling was used to analyze differences between the group with FRD and the group with no dietary restriction. Incidence of chylous ascites was analyzed with a logistic regression model. A longitudinal analysis of the amount of drain fluid was conducted using a mixed effect model with patient-wise random intercepts to account for repeated measurements. The amount of drain fluid after the drain was removed was set to 0, in both the statistical analysis and the plots. Natural cubic splines were used to account for the non-linear trends in drain fluid over time. Time to removal of the drain was analyzed using a Cox regression model. The analyses were done in R (version 3.4.0). *t* test was used for comparison of data between the two groups.

## Results

A total of 161 patients’ data were analyzed. Twenty-three patients were excluded because extended D3 mesenterectomy was not performed, either due to the unavailability of surgeons or due to advanced disease. The remaining 138 patients in the population were divided into the two groups described above; group 1, 39 patients (mean age, 66.07; SD, 10.40; M:F = 15:24) and group 2, 99 patients (mean age, 65.44; SD, 9.85; M:F = 44:55). Three patients originally meant to be in group 2 received fatty food from relatives visiting and were thereby moved to group 1 when this was discovered. They count as 3 of the 39 patients in group 1. All three patients developed chylous ascites.

Sixteen (41.0%) of the thirty-nine patients in group 1 developed chylous ascites according to the parameters described above, while only 1 (1.0%) of the 99 patients in group 2 developed chylous ascites while consuming a FRD (odds ratio = 0.015, *p* < 0.001).

**Fig. 2:**
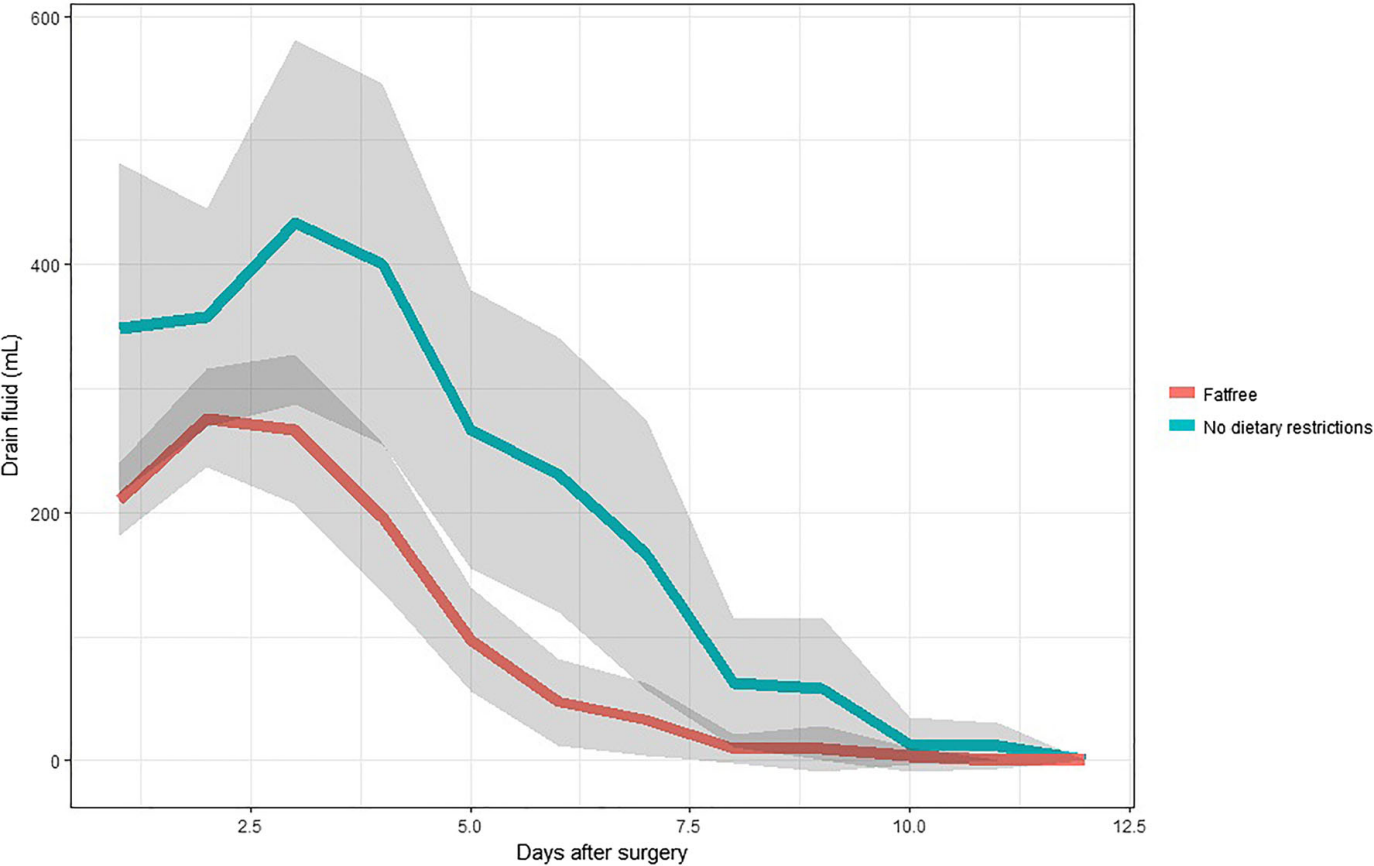
When comparing group 2 (no dietary restriction, green line) with group 3 (FRD, red line) on a group level, there is a significant difference in the amount of drain fluid (*y*-axis). Standard deviation (SD) illustrated as gray areas

**Fig. 3:**
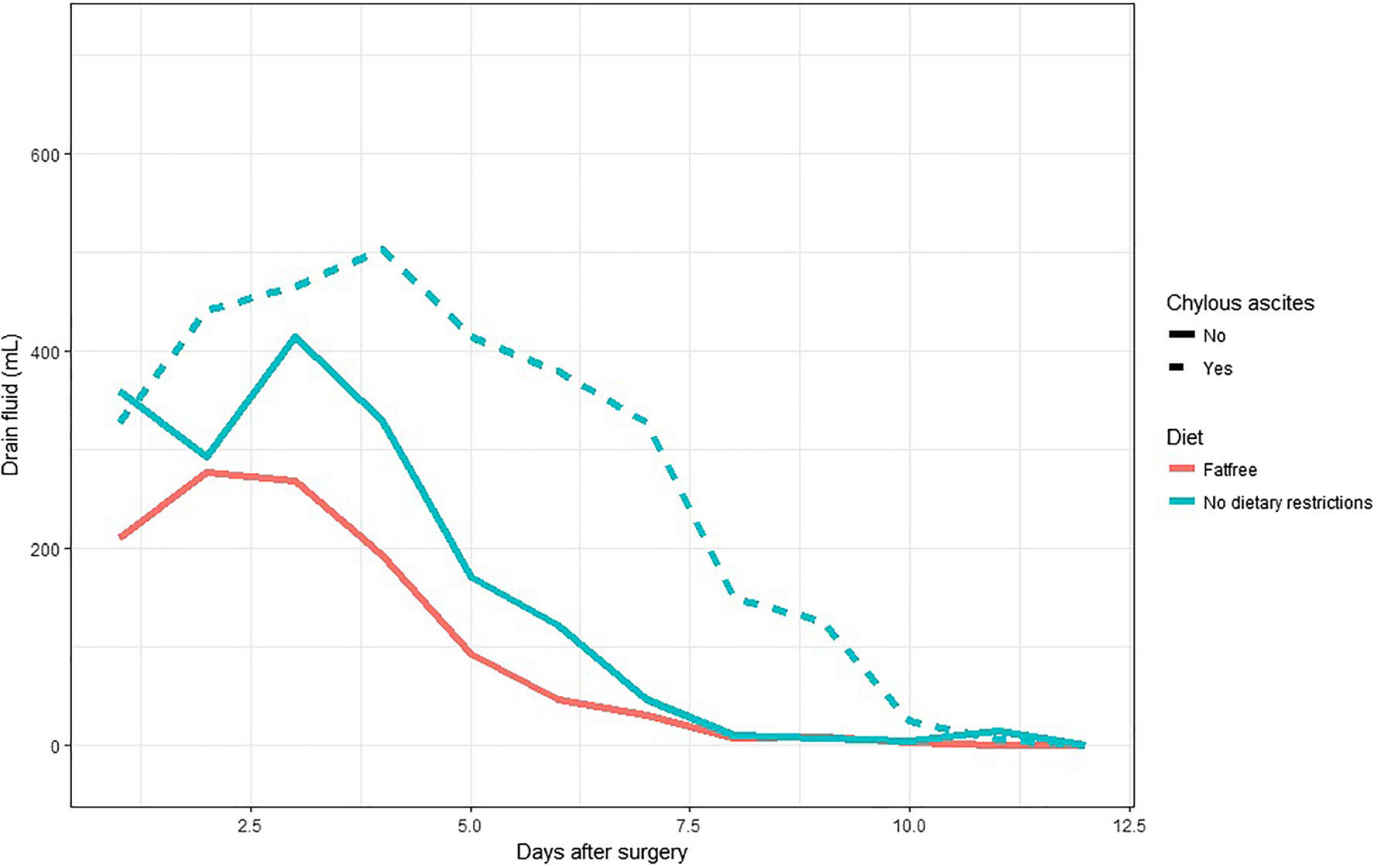
Even when we remove the patients with chylous ascites (green dotted line) from group 2 (no dietary restrictions, remaining patients in group 2 illustrated with whole green line), we see a clear difference in the amount of drain fluid (*y*-axis) compared with group 3 (whole red line, fat-free diet)

When the two groups were compared, the amount of drain discharge was higher in group 1 than in group 2 (Fig. 2; Table 1). The development is not linear as the leakage increases in both groups in the first days before it decreases towards non-significant levels or no leakage. When a regression analysis is done, the average leakage in group 2 is 105 mL/day less than in the group with no dietary restrictions (*p* < 0.001). This makes it highly unlikely to develop chylous ascites after surgery while consuming a FRD. Figure 3 shows the patients in group 1 with no chylous ascites (whole green line) and the patients with chylous ascites (dotted green line) separately. Even when the patients that developed chylous ascites are removed from both groups, the patients with no dietary restrictions have a higher drain output than patients on a FRD (whole red line) (Fig. 3).

**Table 1:** Comparing the amount of drain fluid (mL), cholesterol levels (mmol/L), and triglyceride levels (mmol/L) on the first, third, and fifth postoperative days in the two groups

	Drain fluid (mL)			Cholesterol (mmol/L)			Triglycerides (mmol/L)		
	Day 1	Day 3	Day 5	Day 1	Day 3	Day 5	Day 1	Day 3	Day 5
Non-FRD	357.3 ± 44.2	400.0 ± 72.7	231.3 ± 55.3	1.51 ± 0.25	1.16 ± 0.28	0.96 ± 0.40	1.27 ± 0.41	2.93 ± 0.95	3.65 ± 1.72
FRD	276.2 ± 19.9	196.7 ± 29.9	47.1 ± 17.8	1.64 ± 0.23	1.23 ± 0.10	1.21 ± 0.36	0.86 ± 0.06	0.82 ± 0.06	1.49 ± 0.71

The triglyceride drain fluid levels were lower in the group with fat-reduced diet as compared with the group with no dietary restrictions (Table 1). In group 1, the triglyceride levels are increasing from the first day, whereas in group 2, the triglyceride levels remain low until day 3. After 3 days, the abdominal drain was discontinued for most patients, leaving only those patients with CA in both groups. Regarding cholesterol levels, there is no obvious difference between the two groups (Table 1). The same limitation as with triglyceride levels applies with cholesterol levels with registrations only for the patients that kept the drain beyond the planned 3 days, implying CA.

**Fig. 4:**
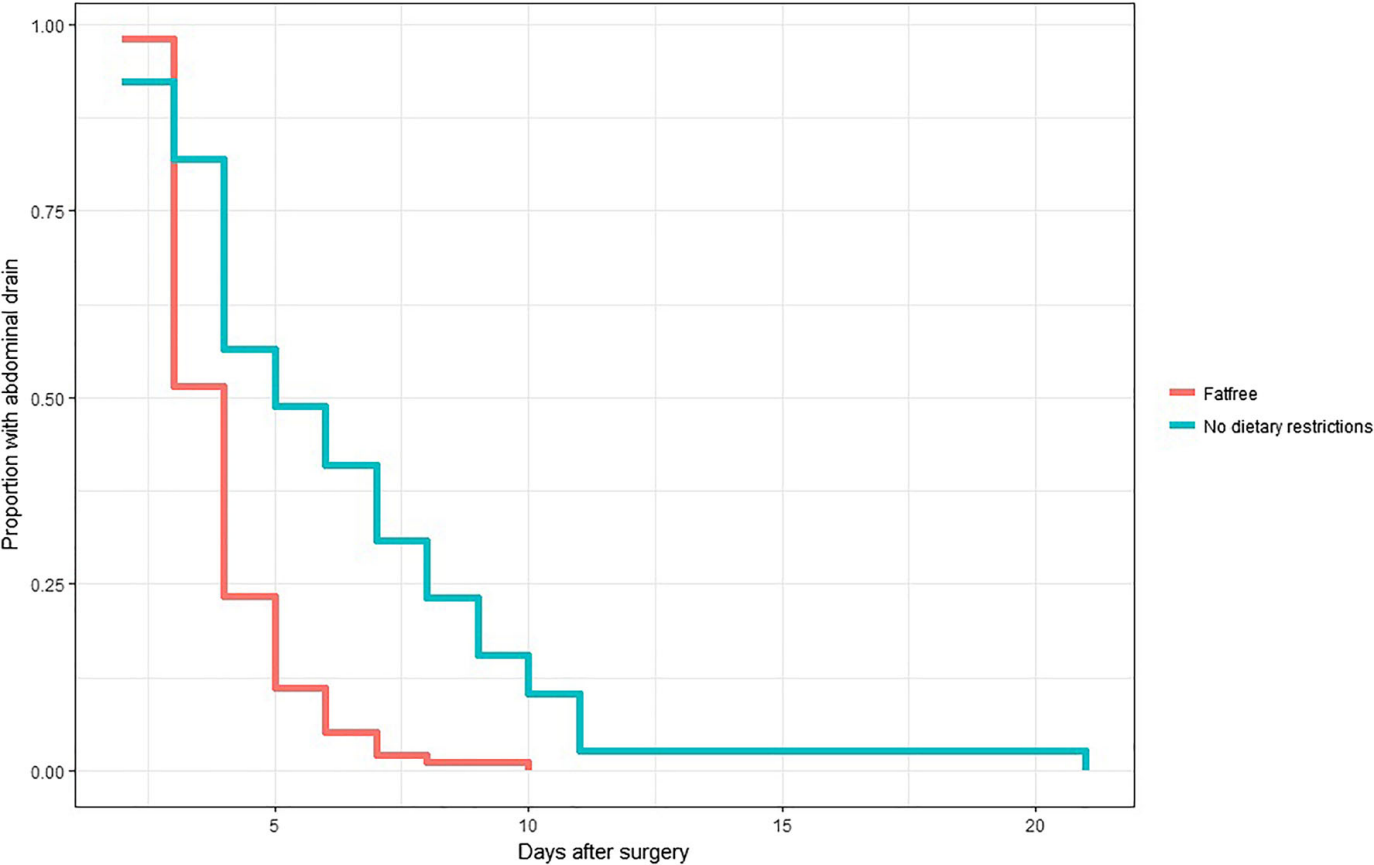
The plot shows how many days after surgery (*x*-axis) the drain is kept in group 2 (no dietary restriction, green line) compared with group 3 (FRD, red line). Proportion of group with abdominal drain along the *y*-axis

We also registered how many days it took before the abdominal drain was removed. This study required that the abdominal drain was kept at least three postoperative days. The drain was removed when there was no chylous ascites detected and the fluid amount was medically and clinically acceptable, a decision made by the surgeon. When comparing the mean number of days the drain was kept, the group with no dietary restrictions (group 1) kept the drain 6.3 days (SD, 3.6) and the group with FRD 3.9 days (SD, 1.3) (Fig. 4). There is a significant difference between the two groups with *p* < 0.001. When estimating the hazard ratio for prolonged drain discharge using a Cox model, we find a ratio of 2.88 (*p* < 0.001), indicating that patients with no dietary restrictions keep the abdominal drain longer compared with patients consuming a FRD, as indicated in Fig. 4.

In group 1, the mean length of hospital stay was 8.9 days (SD 4.9) and in group 2, the mean hospital stay was 7.3 (SD 5.4), but there were no significant differences between the two groups (*p* = 0.19). In both groups, other complications, namely pulmonary embolism, anastomotic leak, deep vein thrombosis, and pneumonia, occurred, but there was no significant difference concerning the occurrence of the total of other complications when comparing the two groups (Fisher exact test, one-tailed *p* = 0.8845).

## Discussion

The cause of chylous leak after surgery performed in the mesentery is thought to be a direct leakage from disrupted lymphatic vessels. Anatomically, the D3 area as defined earlier presumably contains the common lymphatic drainage of the midgut [2]. As the concept of D3 lymphadenectomy is removing more lymph nodes in the root of the mesentery, it seems inevitable that the risk of injuring some of the lymphatic vessels will be higher, thereby increasing the likelihood of chylous ascites. Chyle (a mixture of lymph and chylomicrons absorbed from the small intestine) drains into the cisterna chyli and the thoracic duct. Chylous ascites occurring after bowel surgery is of a different origin when compared with chylous ascites occurring after other types of abdominal surgery due to a separate origin of the chylous fluid. This difference is essential for the proposed treatment/prevention because the lymph flow from the intestines is a function of dietary fat. This implies that a lower intake of fat results in less lymph flow and that the intestinal lymph flow is at its lowest rate after a night of fasting, immediately before the planned surgery. A postoperative FRD will reduce the amount of chyle after surgery, and thereby making chylous ascites less likely. The theory being that this allows for the lymphatic vessels to stay collapsed and hence close. Whether it is the flow of chyle itself or the pressure the chyle exerts on the disrupted lymphatic vessels which causes the chylous ascites when no dietary restrictions are made will only be speculations.

The most important find of this study is that development of CA of intestinal origin seems to be prevented using a routine 3-day postoperative FRD. It is commonly accepted among physicians that chylous ascites occurs due to injury to the cisterna chyli, right and left lumbar lymphatic trunks, as well as the intestinal trunks. Furthermore, the literature states that lymphatic flow through the intestinal trunks increases significantly after the consumption of a heavy meal, something that does not apply to the lumbar trunks. On the other hand, the traditional conservative treatment recommended is the consumption of a FRD. When this does not provide adequate results, TPN is recommended. Reoperation is saved as a last option [5, 7, 12]. The literature contains very few articles dealing with the prevention of chylous ascites. One of these recommends ingestion of sesame oil on the evening before surgery for better identification of large lymphatic vessels at surgery when performing pre-aortal lymphadenectomy, an area not an integral part of the intestinal lymph flow [13]. One can speculate that such a strategy would not be beneficial when operating in the central mesentery, since it could potentially lead to more frequent and voluminous chylous ascites.

In our study, the incidence of chylous ascites after D3 extended mesenterectomy was 41.0%. Apart from the fact that the FRD was introduced, the procedure remained unchanged in 99 patients; only one of them developed minor chylous ascites. After these 99 patients were operated, routine abdominal drainage was abolished. The 3-day FRD is now implemented as a standard prophylactic measure in all patients undergoing D3 right hemicolectomy for cancer in Akershus University Hospital.

The FRD is a non-expensive prophylactic measure, as the food is already available from the kitchen. No special food needed to be introduced, only the attention and care from the nursing staff insuring the right food to the right patient. We also noticed that some patients to whom fat-reduced food was prescribed actually received fatty food from visiting relatives. Some of these patients developed instant chylous ascites, and since they no longer were on a FRD, they were included in the non-FRD group. This observation is worth mentioning as it happened to three of our patients, and the presence of chylous ascites was within 24 h of eating non-FRD. If we do not count these patients in group 1, there is still 13 of 36 patients in the non-FRD group with chylous ascites (36.1%).

A higher abdominal drain output was seen in patients with no dietary restrictions even after patients that developed biochemically proven chylous ascites were removed from the results. This indicates a possible further benefit of a 3-day FRD after gastrointestinal surgical procedures within the mesentery. FRD may also be relevant as a prophylactic measure when performing surgery on other organs with intestinal lymph flow (gastric, colorectal, pancreatic, and small bowel). This requires further studies.

Since the clinical significance of CA may be variable, it is difficult to make a clear conclusion concerning the exact positive effect of the 3-day FRD. However, the FRD is an inexpensive and relatively easy measure to prevent the potentially serious complication of chylous ascites. With an incidence of 41% of CA after D3 extended mesenterectomy, there is reason to believe that some of the patients would develop clinical significant chylous ascites. The rate of other complications did not differ between the two groups, and although not significant, there is a slight tendency to a shorter hospital stay in group 2. Patients’ follow-up were done according to the recommendations in the Norwegian Cancer Program, with the first appointments at 1 and 6 months postoperatively. None of the patients in either group had symptoms of CA at the time of follow-up, making it less likely that chylous ascites occurred after ending the FRD.

The strength of this study is the two interventions. The first being to insert an abdominal drain in patients in group 1 as a diagnostic tool to provide accurate data on the incidence of chylous ascites, and the second to introduce a uniform FRD as the only intervention in group 2. All patients were operated on by only two surgeons adhering to a single surgical technique. All patient meals came from the same institution insuring the same diet. All patients were admitted to the same surgical ward, in this manner reducing the number of staff involved in the postoperative period, tests and diet to a minimum.

The research group did not consider the increased probability to develop chylous ascites when the trial was designed. The relevance of CA was only taken into account as the trial was in progress prompting changes in the protocol. Another limitation is that this is a single-center study with prospective data collection and not a randomized trial, which would surely add to the accuracy of the results. Furthermore, routine use of surgical drains to detect chylous ascites may well have overestimated the clinically relevant incidence, implying that some of the chylous ascites leaks detected would otherwise have gone unnoticed.

Recent literature has presented the lymphatic system of the right colon as a complex three-dimensional structure. It is reflected through the positioning of nodes posterior and anterior to the superior mesenteric vessels according to the crossing pattern of the ileocolic artery to the superior mesenteric vein [2]. Moreover, lymphatic vessels draining directly into central nodes have been demonstrated [11]. A recent clinical study from Japan [14] emphasizes the value of the extent of mesenterectomy through showing significant differences in disease-free survival, as well as the prognostic value of the ileocolic artery crossing pattern in patients with stage III cecum cancer. This implies a possible benefit of this surgical strategy as compared with procedures which do not entail lymphadenectomy posterior to the superior mesenteric vessels [15].

## Conclusions

Chylous ascites is a common postoperative occurrence after right colectomy with extended D3 mesenterectomy with an incidence of 41%. A 3-day routine fat-reduced diet seems to be an effective measure in preventing chylous ascites when operating in the central mesentery.
